# Activation of formyl peptide receptor 1 elicits therapeutic effects against collagen‐induced arthritis

**DOI:** 10.1111/jcmm.16854

**Published:** 2021-08-10

**Authors:** Byunghyun Park, Mingyu Lee, Sang Doo Kim, Yu Sun Jeong, Ji Cheol Kim, Siyoung Yang, Hye Young Kim, Yoe‐Sik Bae

**Affiliations:** ^1^ Department of Biological Sciences Sungkyunkwan University Suwon Korea; ^2^ Department of Health Sciences and Technology Samsung Advanced Institute for Health Sciences and Technology Sungkyunkwan University Seoul Korea; ^3^ Department of Pharmacology Ajou University School of Medicine Suwon Korea; ^4^ Laboratory of Mucosal Immunology Department of Biomedical Sciences Seoul National University College of Medicine Seoul Korea; ^5^ Present address: YK Therapeutics Inc 117, Hwanggeum‐ro, Yangchon‐eup Gimpo Korea

**Keywords:** collagen‐induced arthritis, dendritic cell, formyl peptide receptor, rheumatoid arthritis, T cell

## Abstract

Rheumatoid arthritis (RA) is an autoimmune disorder which shows production of autoantibodies, inflammation, bone erosion, swelling and pain in joints. In this study, we examined the effects of an immune‐modulating peptide, WKYMVm, that is an agonist for formyl peptide receptors (FPRs). Administration of WKYMVm into collagen‐induced arthritis (CIA) mice, an animal model for RA, attenuated paw thickness, clinical scores, production of type II collagen‐specific antibodies and inflammatory cytokines. WKYMVm treatment also decreased the numbers of T_H_1 and T_H_17 cells in the spleens of CIA mice. WKYMVm attenuated T_H_1 and T_H_17 differentiation in a dendritic cell (DC)‐dependent manner. WKYMVm‐induced beneficial effects against CIA and WKYMVm‐attenuated T_H_1 and T_H_17 differentiation were reversed by cyclosporin H but not by WRW4, indicating a crucial role of FPR1. We also found that WKYMVm augmented IL‐10 production from lipopolysaccharide‐stimulated DCs and WKYMVm failed to suppress T_H_1 and T_H_17 differentiation in the presence of anti‐IL‐10 antibody. The therapeutic administration of WKYMVm also elicited beneficial outcome against CIA. Collectively, we demonstrate that WKYMVm stimulation of FPR1 in DCs suppresses the generation of T_H_1 and T_H_17 cells via IL‐10 production, providing novel insight into the function of FPR1 in regulating CIA pathogenesis.

## 1 INTRODUCTION

Rheumatoid arthritis (RA) is a chronic autoimmune disease that affects mainly flexible joints and articular cartilage.^1^ In the pathogenesis of RA, inflammatory cells including mononuclear cells are recruited into the synovium and accelerate the destructive immune cascades.^1^, ^2^, ^3^, ^4^ Consequently, the inflammatory circumstance triggers uncontrolled osteoclast differentiation and fibroblast proliferation, leading to the bone deformities and cartilage erosion within synovial region.^5^, ^6^ Collagen‐induced arthritis (CIA), a representative experimental model for mouse autoimmune arthritis, shares several pathological characteristics with RA such as mononuclear cell infiltration, synovial hyperplasia and cartilage destruction, showing a multifaceted, immunologically complex progress in the disease.^7^ In the centre of RA pathogenesis, dendritic cells (DCs) play crucial roles in activation of CD4 lymphocytes by presenting proper T‐cell receptor stimulatory and co‐stimulatory signalling cues, and context‐dependent cytokines, polarizing them into several subsets of CD4 T cells including T_H_1 and T_H_17.^8^ Meanwhile, tolerogenic DC has immuno‐suppressive properties and sustain peripheral tolerance by preventing excessive lymphocytes activation with anti‐inflammatory surface molecules and cytokines such as TGF‐β and IL‐10.^9^ Since the progress of RA is associated with dysregulated activation of T_H_1 and T_H_17 lymphocytes, it is therefore crucial to identify molecular targets for switching the DC responses to tolerogenic states while suppressing excessive immune activation.

Formyl peptide receptors (FPRs), well‐known chemoattractant receptors for leukocyte recruitment, are expressed in diverse immune system^10^, ^11^ and can regulate immune cell activation and differentiation.^12^ FPRs can recognize a diverse range of agonists that include formyl peptides derived from bacteria or mitochondria and host‐derived agonists (serum amyloid A, LL‐37) and regulate immune cell response in a ligand‐specific manner.^10^, ^13^, ^14^ WKYMVm, a surrogate agonist for FPRs,^15^, ^16^ shows therapeutic effects against several infectious and inflammatory diseases such as polymicrobial sepsis, ulcerative colitis and respiratory disease,^17^, ^18^, ^19^, ^20^, ^21^ implying the important roles of FPRs in immune modulation. Previously, the function of FPRs was investigated in autoimmune arthritis,^22^, ^23^ and it was known that serum amyloid A, an another endogenous FPR2 agonist, mediates synovial hyperplasia and angiogenesis via FPR2 of synovial fibroblasts during progress of RA.^24^, ^25^ However, the function of FPRs in adaptive immunity remains unclear in autoimmune disease. In this study, we investigated the roles of FPR in autoimmune disease with a well‐known FPR agonist WKYMVm in a CIA mouse model by focusing on DC‐mediated CD4 T‐cell differentiation.

## 2 MATERIALS AND METHODS

### 2.1 CIA mouse model

All experiments involving animals received the approval of the Institutional Review Committee for Animal Care and Use at Sungkyunkwan University (Suwon, Korea). 8‐ to 12‐week‐old DBA/1J male mice were purchased from Orient Bio Inc. (Seongnam, Korea). Mice were immunized by bovine type II collagen (CII, Chondrex, Redmond, WA, USA) with complete Freund's adjuvant (Chondrex, Redmond, WA, USA), and secondary boosting was conducted 21 days after the first immunization with CII and incomplete Freund's adjuvant (Chondrex, Redmond, WA, USA). CIA mice were monitored for paw thickness, and clinical scores were measured from the day of secondary boosting. Front and hind paws of each side were measured by using a caliper and scored according to a published protocol^26^ and each score from individuals were combined. Vehicle (1× phosphate‐buffered saline) or WKYMVm, synthesized by Anygen (Gwangju, Korea) with a purity >99.6%, was subcutaneously injected into the CIA mice model daily following the secondary boosting. Cyclosporin H (CsH) (Enzo Life Sciences, Farmingdale, New York, USA) and WRW4 (Anygen, Gwangju, Korea) were subcutaneously injected 30 min before WKYMVm injection. For the therapeutic administration, WKYMVm was subcutaneously injected into the CIA mice model daily after onset of the clinical signs of CIA. After monitoring CIA, mice were sacrificed at the CIA peak after secondary boosting for analysis.

### 2.2 Enzyme‐linked immunosorbent assay (ELISA)

The levels of IgG1 and IgG2a reactive to immunized collagen in the peripheral blood serum were determined by using a mouse anti‐bovine CII IgG1 and IgG2a antibody assay kit with tetramethylbenzidine (TMB) substrate (Chondrex, Redmond, WA, USA). Cytokine ELISA and antibody detection were carried out according to the manufacturer's instructions.

### 2.3 Histology of arthritic joints

One leg was randomly selected from each mouse and dissected for histology. The knee joints were collected and fixed in 4% paraformaldehyde solution. Fixed joints were decalcified in Decalcifying Solution Lite (Sigma‐Aldrich, St. Louis, MO, USA) for 6 h and embedded in paraffin. Joint tissues were sectioned by a microtome, and sections (5 μm) had been hydrated in 100%, 90%, 70%, and 50% ethanol in distilled water (DW) for 5 min each. Hydrated samples were stained with haematoxylin and eosin (H&E) (Sigma‐Aldrich, St. Louis, MO, USA) for morphological analysis and measurement of the infiltration of immune cells, or safranin O and fast green (Sigma‐Aldrich, St. Louis, MO, USA) for determining cartilage damage. Stained samples were dehydrated in 50%, 70%, 90% and 100% ethanol and xylene and mounted by using balsam. They were observed by using DM750 microscope (Leica, Wetzlar, Germany).

### 2.4 Intracellular cytokine staining and flow cytometry

For the detection of intracellular cytokines, cells were reactivated by PMA (50 ng/ml) and ionomycin (500 ng/ml) (Sigma‐Aldrich) with protein transport inhibitor (Thermo Fisher Scientific, Waltham, MA, USA) for 5 h. Cells were blocked by anti‐mouse CD16/32 antibodies before surface staining. Surface proteins expressed on cells were stained with fluorescence‐conjugated antibodies diluted in FACS buffer (1× phosphate‐buffered saline with 0.5% bovine serum albumin) for 30 min. Intracellular cytokine staining was performed using intracellular fixation and permeabilization buffer set (Thermo Fisher Scientific, Waltham, MA, USA) following the manufacturer's recommended protocol. The anti‐mouse CD4‐PerCP‐Cy7, IL‐17A‐APC, IL‐17A‐PE, IFNγ‐APC and IFNγ‐PE fluorescence‐conjugated antibodies for flow cytometry were purchased from Thermo Fisher Scientific (Waltham, MA, USA). After staining, cells were washed and acquired on FACS Canto II (BD Biosciences, Franklin Lakes, NJ, USA). To figure out cytokine‐producing CD4 T cells, lymphocytes were gated from FSC‐A and SSC‐A dot plot. Next, single cells were gated from FSC‐H and FSC‐A. CD4^+^ cells were gated from CD4‐Percp‐cy5.5 and FSC‐A dot plot. And then, intracellular cytokines in CD4 T cells were measured. Data were analysed by using FlowJo software (FlowJo, LLC, Ashland, OR, USA).

### 2.5 In vitro differentiation of T_H_1 or T_H_17 cells

C57BL/6 mice were purchased from Orient Bio Inc. (Seongnam, Korea). Mouse naïve CD4 T cells were isolated from the splenocytes of 8– 12‐week‐old C57BL/6 mice by using MagniSort™ mouse CD4 naive T‐cell enrichment kit (Thermo Fisher Scientific, Waltham, MA, USA). Naïve CD4 T cells were stimulated by anti‐CD3e (2 μg/ml) and anti‐CD28 (2 μg/ml) in the presence of mIL‐12p70 (10 ng/ml) and anti‐mIL‐4 (10 μg/ml) for T_H_1 cell differentiation, and mTGFβ (3 ng/ml), mIL‐6 (20 ng/ml) and mIL‐23 (20 ng/ml) for T_H_17 cell differentiation. Recombinant cytokines were purchased from Thermo Fisher Scientific (Waltham, MA, USA). Cells were cultured with vehicle or 1 μM WKYMVm in 96‐well plates for 4 days in RPMI 1640 (Welgene, Gyeongsan, Korea) containing 10% foetal bovine serum, 2 mM L‐glutamine, 0.05 mM 2‐mercaptoethanol and 100 U/ml penicillin and streptomycin. T_H_1 and T_H_17 cell differentiation were measured after intracellular cytokine staining by flow cytometry. Differentiated T cells were reactivated by PMA and ionomycin for 24 h, and cytokines in the supernatant were measured by using ELISA kits (Thermo Fisher Scientific, Waltham, MA, USA).

### 2.6 Ex vivo collagen stimulation

Splenocytes were isolated from CIA mice at 35 days after immunization. Cells were stimulated by CII (50 μg/ml) with vehicle or 1 μM WKYMVm and cultured in 48‐well plates for 3 days in RPMI 1640 (Welgene, Gyeongsan, Korea) containing 10% foetal bovine serum, 2 mM L‐glutamine, 0.05 mM 2‐mercaptoethanol and 100 U/ml penicillin and streptomycin. The cytokines in the supernatant were measured by using ELISA kits (Thermo Fisher Scientific, Waltham, MA, USA) according to the manufacturer's instructions.

### 2.7 Generation of bone marrow‐derived DCs (BMDCs) and maturation

Mouse bone marrow cells were isolated from 8‐ to 12‐week‐old C57BL/6 or DBA/1J mice and differentiated for 6 days with 20 ng/ml GM‐CSF in RPMI 1640 containing 10% foetal bovine serum, 2 mM L‐glutamine, 0.05 mM 2‐mercaptoethanol and 100 U/ml penicillin and streptomycin. After 2 days, the medium changed to fresh medium including 20 ng/ml GM‐CSF. At 4 days after the start of differentiation, additional fresh medium containing 20 ng/ml GM‐CSF was added to the culture. 6 days after initiation of differentiation, differentiated BMDCs were collected and matured with 100 ng/ml LPS for 24 h with vehicle or 1 μM WKYMVm. 1 μM CsH or 10 μM WRW4 was added at 15 min before WKYMVm treatment. Cytokines in the supernatant were measured by using ELISA kits (Thermo Fisher Scientific, Waltham, MA, USA).

### 2.8 Co‐culture of BMDCs with naïve CD4 T cells

C57BL/6 OT‐II transgenic mice were kindly provided by Prof. Yong‐Soo Bae from Sungkyunkwan University. Naïve CD4 T cells isolated from the spleens of 8‐ to 12‐week‐old C57BL/6‐based OT‐II T‐cell receptor (TCR) transgenic mice or DBA/1J mice with CIA. BMDCs were matured with 100 ng/ml LPS for 24 h and treated with 1 μg/ml ovalbumin 1 h before preparation. Mature BMDCs and naïve CD4 T cells were co‐cultured at a 1:5 ratio with vehicle and 1 μM WKYMVm for 5 days. 1 μM CsH or 10 μM WRW4 was added 15 min before WKYMVm treatment. IL‐10 neutralizing antibodies (R&D systems, Minneapolis, MN, USA) (10 μg/ml) were added in the absence or presence of WKYMVm in the co‐culture of BMDCs with naïve CD4 T cells. The cells were cultured in RPMI 1640 (Welgene, Gyeongsan, Korea) containing 10% foetal bovine serum, 2 mM L‐glutamine, 0.05 mM 2‐mercaptoethanol and 100 U/ml penicillin and streptomycin. For T_H_17 cell differentiation, recombinant mTGFβ (3 ng/ml), mIL‐6 (20 ng/ml) and mIL‐23 (20 ng/ml) were added to the culture. IFNγ‐ and IL‐17A‐producing CD4 T‐cell population were measured by flow cytometry. Cells were also reactivated by 50 ng/ml PMA and 500 ng/ml ionomycin for 24 h, and cytokines in supernatant were measured by using ELISA kits (Thermo Fisher Scientific, Waltham, MA, USA).

### 2.9 Statistical analysis

All results are expressed as the mean ± SEM for the data obtained from the indicated number of experiments. Statistical analysis was performed using Student's *t* test or two‐way ANOVA. A *p* value ≤ 0.05 was considered statistically significant.

## 3 RESULTS

### 3.1 An immune‐modulating peptide, WKYMVm, elicits beneficial effects against CIA

**FIGURE 1:**
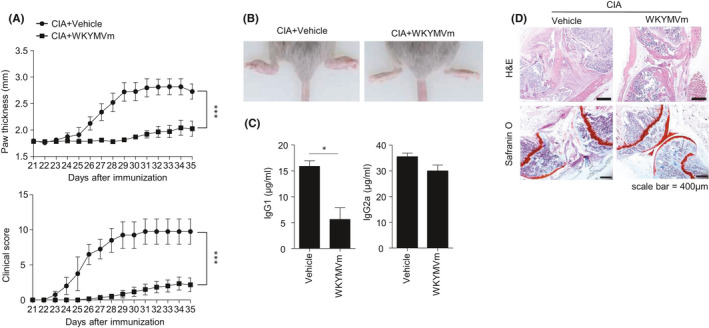
WKYMVm administration elicits beneficial effects against CIA. (A‐D) Vehicle or WKYMVm (4 mg/kg) was subcutaneously injected daily into CIA mice from secondary boosting at day 21. Mice were sacrificed on day 35. (A) Paw thickness (top) and clinical scores (bottom) of CIA mice were monitored during the indicated period. (B) Paw swelling of the two groups compared at the day of sacrifice. Representative images are shown. (C) CII reactive IgG1 and IgG2a were measured in peripheral blood serum from each group of mice. (D) Joint tissue sections were stained with H&E and safranin O (×40). Representative figures are shown. Data are expressed as the mean ± SEM (*n* = 4–6 per group). *p* values were calculated by two‐way ANOVA (A) or Student's *t* test (C). *p < 0.05, ***p < 0.001

First, we examined the effects of WKYMVm, a surrogate agonist for FPRs, on CIA according to a previous report.^27^ CIA mice with vehicle showed markedly increased paw thickness and clinical scores from day 26, which were sustained up to 35 days after immunization (Figure 1A). However, administration of WKYMVm in CIA mice significantly attenuated the clinical signs of CIA, showing decreased paw swelling compared to the vehicle‐treated arthritic group (Figure 1A, B). Because the levels of collagen‐specific IgG1 and IgG2a correlate well with the development of arthritis,^28^ we also examined collagen‐specific IgG1 and IgG2a production in CIA mice. Correlating with the attenuation of the clinical signs of CIA, the WKYMVm‐injected CIA group showed significantly decreased IgG1 levels compared to vehicle‐injected CIA mice (Figure 1C left). IgG2a levels were also slightly decreased by WKYMVm (Figure 1C right). Histological analysis of inflamed knees through H&E staining revealed that CIA mice showed severe joint destruction with heavily infiltrated immune cells within synovial regions, but WKYMVm administration elicited diminished joint damage and immune cell infiltration (Figure 1D top). Safranin O staining analysis revealed that CIA mice showed cartilage loss, which was markedly recovered upon WKYMVm administration (Figure 1D bottom). Collectively, the results suggest that WKYMVm shows beneficial effects against CIA.

### 3.2 FPR1 mediates WKYMVm‐induced beneficial effects against CIA

**FIGURE 2:**
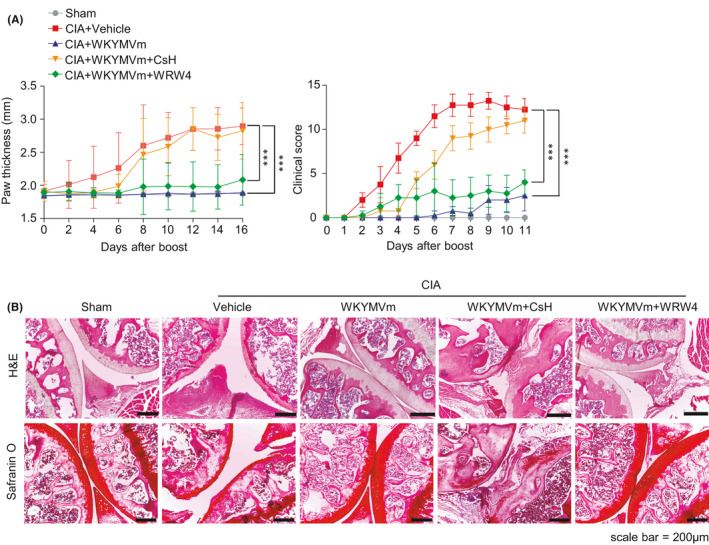
The effect of WKYMVm on CIA is mediated by FPR1. (A,B) Vehicle or WKYMVm (4 mg/kg) was subcutaneously injected daily into CIA mice starting from the day of secondary boosting. CsH (4 mg/kg) or WRW4 (4 mg/kg) was administrated by the same injection route at 30 min before WKYMVm injection. (A) Paw thickness of CIA mice was monitored during the indicated period. (B) Joint tissue sections were stained with H&E (top) or safranin O (bottom) (×100). Mice were sacrificed on day 43. Data are expressed as the mean ± SEM (*n* = 4 per group). *p* values were calculated by two‐way ANOVA (A) ***p < 0.001

Previous reports demonstrated that WKYMVm acts on three different FPR members (FPR1, FPR2 and FPR3) in human leukocytes and at least two FPR members (FPR1 and FPR2) in mouse leukocytes.^14^, ^29^ In this study, we examined which FPR subtype is involved in the beneficial effects of WKYMVm against CIA by using FPR1 or FPR2 antagonists, CsH or WRW4, respectively. Administration of CsH, an FPR1 antagonist, blocked WKYMVm‐elicited beneficial effects against CIA, showing increased paw thickness compared to the WKYMVm‐alone group (Figure 2A). However, WRW4, an FPR2 antagonist, did not affect WKYMVm‐induced beneficial effects against CIA (Figure 2A). Histological analysis showed that WKYMVm‐induced joint damage reduction was blocked by CsH but not by WRW4 (Figure 2B). Through H&E and safranin O staining, we also found that WKYMVm‐induced cartilage restoration and inhibition of immune cell infiltration were blocked by CsH but not by WRW4 (Figure 2B). Taken together, our results suggest that WKYMVm‐induced beneficial effects against CIA are mediated by FPR1 but not by FPR2.

### 3.3 WKYMVm inhibits IFN‐γ‐ or IL‐17A‐producing CD4 T cells via FPR1 in CIA mice

**FIGURE 3:**
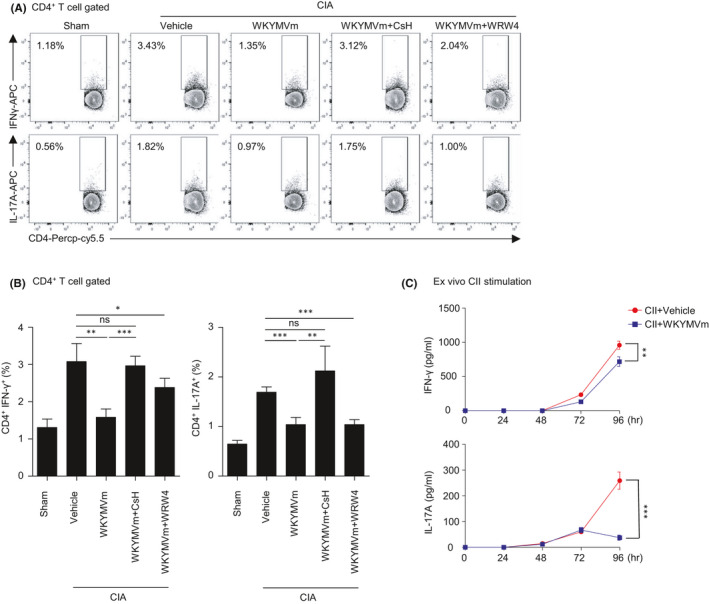
WKYMVm administration decreases IFNγ‐ or IL‐17A‐producing CD4 T cells in CIA mice. (A,B) Vehicle or WKYMVm (4 mg/kg) was subcutaneously injected daily into CIA mice starting from the day of secondary boosting at day 21. CsH (4 mg/kg) or WRW4 (4 mg/kg) was administrated by the same injection route at 30 min before WKYMVm injection. CD4 T‐cell populations of mice were analysed by flow cytometry. (A) IL‐17A‐ or IFN‐γ‐producing CD4 T‐cell populations in the splenocytes from each group were compared by flow cytometry. Representative dot plot data for each group are shown (A). Splenocytes had been activated by PMA (50 ng/ml) and ionomycin (500 ng/ml) with protein transport inhibitor. Indicated population of CD4 T cells were compared among the groups. (B) IFN‐γ‐ and IL‐17A‐producing CD4 T‐cell populations were analysed. (C) Splenocytes from CIA mice were stimulated by CII. Cytokines in supernatant at the indicated time points were measured by ELISA. Data are expressed as the mean ± SEM (*n* = 4 for A, B, C). *p* values were calculated by Student's *t* test (B) or two‐way ANOVA (C). *p < 0.05, **p < 0.01, ***p < 0.001. ns, not significant

Previously, several cytokines were reported to mediate the pathogenesis of RA.^30^ Among these, IFN‐γ and IL‐17 are the major contributors in RA progression.^14^, ^15^, ^31^ The major sources of these cytokines are effector CD4 T cells differentiated into T_H_1 and T_H_17. IFN‐γ and IL‐17A are known to induce the expression of cell‐to‐cell interaction molecules and activate fibroblast‐like synoviocytes to mediate inflammation in the synovium.^32^ In this study, we observed that the establishment of CIA in mice could augment the levels of IFN‐γ^+^ CD4 T cells and IL‐17A^+^ CD4 T cells (Figure 3A,B). We then examined the effects of WKYMVm on the IFN‐γ^+^ or IL‐17A^+^ CD4 T‐cell populations in CIA mice. As shown in Figure 3A, WKYMVm administration suppressed the formation of IFN‐γ^+^ or IL‐17A^+^ CD4 T cells in CIA mice. The results suggest that WKYMVm administration may decrease the clinical signs of CIA by downregulating T_H_1 and T_H_17 cells. We also found that WKYMVm‐induced decreases of IFN‐γ^+^ or IL‐17A^+^ CD4 T cells in CIA mice were also significantly blocked by CsH but not by WRW4 (Figure 3A,B), suggesting that FPR1 plays a role in the decrease of T_H_1 and T_H_17 cells by WKYMVm. To confirm the effects of WKYMVm on the suppression of T_H_1 and T_H_17 in CIA mice, we additionally conducted ex vivo experiments with CIA mice. Splenocytes isolated from CIA mice were restimulated by CII and simultaneously treated with vehicle or WKYMVm during activation. WKYMVm treatment significantly decreased IL‐17 and IFN‐γ production (Figure 3C). Taken together, WKYMVm effectively blocked CII specific T_H_1‐ and T_H_17‐mediated immune reactions and this effect was mediated by FPR1.

### 3.4 WKYMVm‐induced decrease of T_H_1 and T_H_17 cells is dependent on FPR1 expressed on DCs

**FIGURE 4:**
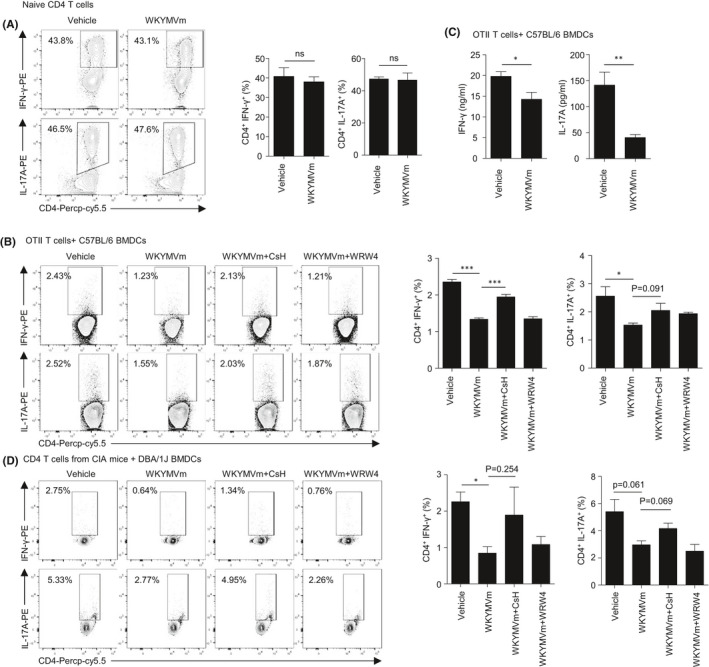
Suppression of T_H_1 and T_H_17 differentiation by WKYMVm is dependent on FPR1 of DCs. (A) Naïve CD4 T cells were isolated from the splenocytes of WT C57BL/6 mice and differentiated in T_H_1‐ and T_H_17‐polarizing conditions with vehicle or 1 μM WKYMVm for 4 days. IFN‐γ‐ or IL‐17A‐producing CD4 T‐cell populations in each group were analysed by flow cytometry. (B, C) Naïve CD4 T cells isolated from the splenocytes of OT‐II TCR transgenic mice and matured BMDCs stimulated by LPS (100 ng/ml) with OVA were co‐cultured for 5 days with vehicle or 1 μM WKYMVm. Each group of co‐cultured cells was polarized into T_H_1 or T_H_17. (B) 1 μM CsH or 10 μM WRW4 was treated at 15 min before WKYMVm administration. IFN‐γ‐ or IL‐17A‐producing CD4 T‐cell populations in each group were measured by flow cytometry. (C) Vehicle‐ or WKYMVm‐treated cells were reactivated by PMA (50 ng/ml) and ionomycin (500 ng/ml) for 24 h. IFN‐γ and IL‐17A in the supernatant were measured by ELISA. (D) Naive CD4 T cells isolated from CIA mice and matured BMDCs from DBA/1J mice stimulated by LPS with CII were co‐cultured for 5 days with vehicle or 1 μM WKYMVm. Each group of co‐cultured cells was polarized into T_H_1 or T_H_17. CsH and WRW4 were treated the same as in (B). All dot plot figures are representative. Data are expressed as the mean ± SEM (*n* = 4 for A, B, D, *n* = 3 for C). *p* values were calculated by Student's *t* test. *p < 0.05, **p < 0.01, ***p < 0.001

Since WKYMVm administration elicited beneficial effects against CIA by downregulating T_H_1 cells in the spleen (Figures 1 and 3A), we investigated whether WKYMVm can suppress the differentiation of T_H_1 cells from naïve CD4 T cells. We differentiated mouse naïve CD4 T cells into T_H_1 cells in the absence or presence of WKYMVm. T_H_1 cell differentiation from naïve CD4 T cells was not directly affected by WKYMVm (Figure 4A). Since a previous report demonstrated that DCs mediate T‐cell differentiation by presenting stimulatory signals and antigens to T cells,^8^ we investigated whether WKYMVm decreases the cellular differentiation of naïve CD4 T cells into T_H_1 or T_H_17 cells by regulating DC activity. To figure this out, we co‐cultured OTII T cells with BMDCs in the absence or presence of WKYMVm. Unlike the failure of WKYMVm to directly affect T_H_1 cell differentiation, WKYMVm suppressed T_H_1 cell differentiation in the presence of BMDCs (Figure 4B). Co‐culture of OTII T cells with BMDCs generated IFN‐γ^+^ CD4 T cells (2.36 ± 0.14%), while WKYMVm decreased generation (1.33 ± 0.07%) (Figure 4B). The results suggest that WKYMVm indirectly suppresses T_H_1 cell differentiation through acting on the DCs. We next investigated which isoform of FPR is involved in WKYMVm‐induced T_H_1 suppression using an FPR1‐selective antagonist, CsH and an FPR2‐selective antagonist, WRW4. WKYMVm‐induced suppression of the CD4^+^ IFN‐γ^+^ cell population was recovered by CsH treatment but not by WRW4 (Figure 4B). The results suggest that WKYMVm‐induced the suppression of DC‐mediated T_H_1 cell differentiation is mediated by FPR1 but not by FPR2.

Our finding that WKYMVm administration decreased T_H_17 cells in CIA mice led us to examine the effects of WKYMVm on T_H_17 cell generation in vitro. We differentiated mouse naïve CD4 T cells into T_H_17 cells in the absence or presence of WKYMVm. T_H_17 cell differentiation from naïve CD4 T cells was not directly affected by WKYMVm (Figure 4A), suggesting that WKYMVm fails to directly inhibit T_H_17 cell differentiation. We next investigated the effects of WKYMVm on T_H_17 cell generation in the presence of DCs. Similar to T_H_1 cell generation, WKYMVm significantly decreased CD4^+^ IL‐17A^+^ cells in the presence of DCs (Figure 4B). The roles of FPR1 or FPR2 on the WKYMVm‐induced suppression of T_H_17 cells in the presence of DCs were tested using CsH or WRW4. Like T_H_1 cell generation, suppression of T_H_17 cell generation by WKYMVm in the presence of DCs was recovered by CsH treatment but not by WRW4 (Figure 4B), suggesting a crucial role of FPR1. Consistent with the flow cytometry results, co‐cultured cells activated by PMA and ionomycin produce less IFN‐γ and IL‐17A in WKYMVm‐treated cells compared to vehicle‐treated cells (Figure 4C). To further investigate whether the change in T‐cell differentiation was also controlled by DCs presenting CII, BMDCs matured by LPS plus CII were co‐cultured with CD4 T cells from CIA mice which were sensitized to CII. As in the previous results, the generation of T_H_1 and T_H_17 was also suppressed by WKYMVm in co‐culture conditions, showing an FPR1 dependency (Figure 4D). In conclusion, WKYMVm suppresses T_H_1 and T_H_17 cell differentiation in the presence of DCs by working on FPR1 expressed on the surface of DCs, affecting the interaction between the DCs and CD4 T cells.

### 3.5 WKYMVm further increases IL‐10 production from LPS‐stimulated DCs which has a role in suppressing T‐cell differentiation

**FIGURE 5:**
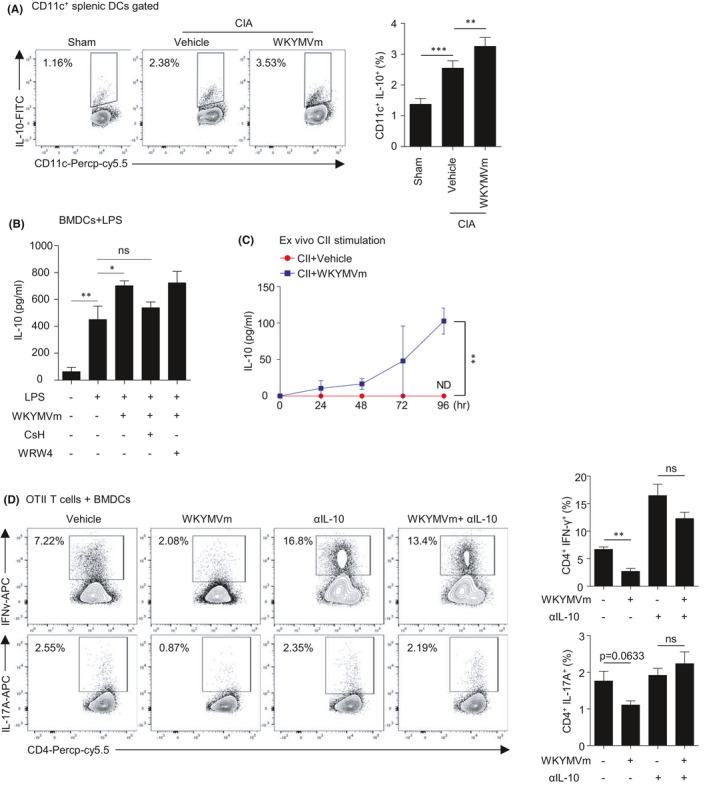
WKYMVm further increases IL‐10 from DCs consequently suppressing T_H_1 and T_H_17 differentiation. (A) CIA was initiated in DBA1/J mice, and vehicle or WKYMVm (4 mg/kg) was injected daily after secondary boosting at day 21. Mice were sacrificed at day 43. IL‐10‐producing CD11c^+^ DCs were gated in the splenocytes of CIA mice. Representative dot plots are shown in the left panel. (B) BMDCs were matured by 100 ng/ml LPS with vehicle or 1 μM WKYMVm for 24 h in the absence or presence of 1 μM CsH, 10 μM WRW4. Levels of IL‐10 in the supernatant were measured by ELISA (B). (C) Splenocytes from CIA mice were ex vivo stimulated by 50 μg/ml CII. IL‐10 was measured by ELISA at the indicated time after stimulation. (D) Naïve CD4 T cells isolated from the splenocytes of OT‐II TCR transgenic mice and BMDCs were co‐cultured for 5 days with vehicle or 1 μM WKYMVm. 10 μg/ml IL‐10 neutralizing antibodies were administered to each group as indicated. Differentiated T_H_1 and T_H_17 cells were analysed by flow cytometry. Representative dot plot data are shown. Data are expressed as the mean ± SEM (*n* = 3–4 for A right, B, C, D right). *p* values were calculated by Student's *t* test (A right, B, D right) or two‐way ANOVA (C). *p < 0.05, **p < 0.01, ***p < 0.001. ns, not significant

DCs are matured in pathogenic conditions, and mature DCs produce several cytokines, present antigens and provide stimulatory signals to T cells.^8^ Mature DCs express high levels of surface molecules such as major histocompatibility complex (MHC) and CD80/86 which have a role in DC‐to‐cell interaction with T cells.^8^ Depending on the surrounding environment, DCs can be stimulatory or regulatory, which may stimulate or suppress T‐cell activation, respectively, leading to polarization of T cells into various effector or regulatory subtypes.^9^ Since we found that WKYMVm suppresses T_H_1 and T_H_17 cell generation in the presence of DCs, we examined whether WKYMVm can affect regulatory cytokine production in CIA mice. And we found that administration of WKYMVm significantly increased IL‐10 producing DCs (Figure 5A). We then tested the effects of WKYMVm on the production of IL‐10 from mature DCs. Being matured by LPS, DCs produced several cytokines such as IL‐10, IL‐6, IL‐12 and IL‐1β. Addition of WKYMVm significantly increased the production of IL‐10 from mature DCs, which was inhibited by CsH but not by WRW4 (Figure 5B), suggesting a crucial role of FPR1. However, WKYMVm did not affect the levels of IL‐6, TNFα, IL‐12 and IL‐1β (data not shown). Furthermore, in the ex vivo stimulation of CII, WKYMVm significantly increased IL‐10 from the splenocytes of CIA mice (Figure 5C). Thus, we next focused on the expected function of IL‐10 from DCs to regulate T‐cell differentiation in vitro.

Previously, it was reported that IL‐10 produced from DCs can suppress T‐cell expansion.^9^, ^33^, ^34^ Here, we aimed to see whether IL‐10 from DCs mediates the suppression of T‐cell differentiation by WKYMVm. For this, we examined the suppression of T_H_1 and T_H_17 cells in the presence of IL‐10‐neutralizing antibody in naïve CD4 T‐cell/DC co‐culture conditions for determining whether the effect of WKYMVm on T cells can be attributed to IL‐10. In our experiment, we found that the neutralization of IL‐10 blocked the suppressing effect of WKYMVm on the generation of CD4^+^ IFN‐γ^+^ cells (Figure 5D top). WKYMVm also failed to suppress the generation of CD4^+^IL‐17^+^ cells in the presence of anti‐IL‐10 antibody (Figure 5D bottom). These results suggest that IL‐10 plays an essential role in the suppression of T_H_1 and T_H_17 cell differentiation by WKYMVm. Taken together, WKYMVm binds to FPR1 expressed on mature DCs and augments IL‐10 secretion to control T_H_1 and T_H_17 differentiation that occurs during the development of CIA.

### 3.6 WKYMVm shows therapeutic effects in experimental CIA

**FIGURE 6:**
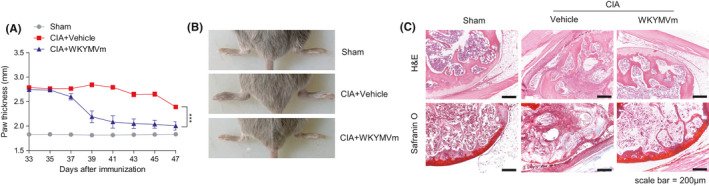
WKYMVm elicits therapeutic effects against CIA. (A‐C) Vehicle or WKYMVm (4 mg/kg) was subcutaneously injected daily into CIA mice from onset of the clinical signs of CIA at 33 days after immunization. Mice were sacrificed on day 47. (A) Paw thickness of CIA mice was monitored during the indicated period. (B) Paw swelling of the two groups compared at the day of sacrifice. Representative images are shown. (C) Joint tissue sections were stained with H&E (top) and safranin O (bottom) (×100). Representative figures are shown. Data are expressed as the mean ± SEM (*n* = 6 per group). *p* values were calculated by two‐way ANOVA (A) ***p < 0.001

We also examined whether WKYMVm shows therapeutic effects against CIA. For this, we administered vehicle or WKYMVm daily after onset of the clinical signs of CIA. As shown in Figure 6A,B, WKYMVm significantly suppressed paw thickness and swelling in therapeutic experimental CIA model. Histological analysis through H&E staining revealed that therapeutic administration of WKYMVm‐elicited decrease of joint damage and immune cell infiltration (Figure 6C top). The therapeutic administration of WKYMVm also decreased cartilage destruction, which was monitored by Safranin O staining analysis (Figure 6C bottom). The results suggest that WKYMVm elicits therapeutic effects against CIA.

## 4 DISCUSSION

In this study, we found that that the immune‐modulating peptide WKYMVm attenuates CIA, resulting in decreased paw thickness, clinical scores and auto‐antibody production (Figure 1). Since administration of FPR agonist WKYMVm suppressed the expansion of splenic T_H_1 and T_H_17 cells in vivo (Figure 3), we speculated that the effects of WKYMVm might be due to direct effects on CD4 T‐cell differentiation of CIA mice. However, WKYMVm failed to affect the cellular differentiation of naïve CD4 T cells into T_H_1 or T_H_17 cells (Figure 4A). Since WKYMVm suppressed the expansion of T_H_1 and T_H_17 cells ex vivo (Figure 3C), we then examined the indirect effects of WKYMVm on cellular differentiation of naïve CD4 T cells. WKYMVm suppressed T_H_1 and T_H_17 cell expansion in the presence of DCs (Figure 4B). Based on our results, we suggest that WKYMVm elicits anti‐CIA effects by suppressing T_H_1 and T_H_17 cell generation, which can be mediated by DC activity.

DCs can be classified into two distinct subsets, the stimulatory and regulatory (or tolerogenic) types.^9^ Regulatory DCs produce IL‐10, which show suppressive responses against active immune responses.^9^ In this study, we attempted to test the effects of WKYMVm on the stimulatory and tolerogenic phenotype of DCs. Although WKYMVm failed to suppress the expression of proteins that provide stimulatory signals such as the CD40 ligand (CD40L), MHC II, CD80/86 (data not shown) and any other cytokines, WKYMVm augmented the production of IL‐10 in response to LPS from DCs (Figure 5B). Since IL‐10 is the representative cytokine produced by regulatory DCs,^9^ and IL‐10 produced from DCs can suppress T‐cell expansion,^33^, ^34^ our results suggest that WKYMVm stimulates the generation of regulatory DCs. In a previous report, we demonstrated that WKYMVm inhibits human monocyte‐derived DC maturation caused by LPS, showing a decrease of IL‐12, decrease of CD86/HLA‐DR and decrease of allostimulatory activity.^35^ Collectively from our previous report and current findings, we suggest that WKYMVm may have more complex effects on DC maturation and differentiation in human monocyte‐derived DCs and mouse BMDCs.

On the functional roles of FPR members in RA, we previously demonstrated that a novel peptide acting on FPR2, scolopendrasin IX, shows therapeutic effects against K/BxN serum‐induced RA.^23^ Another previous report demonstrated that FPR signalling initiated by Cpd43, a dual agonist for FPR1 and FPR2, makes CD4 T cells more apoptotic and inhibits the proliferation of fibroblast‐like synoviocytes, then attenuating the CIA mouse RA model via FPR2.^36^ The functional role of FPR2 in the regulation of RA pathogenesis was also demonstrated by showing that deletion of annexin A1, an endogenous FPR2 agonist, exacerbates arthritis severity in K/BxN serum‐injected mice.^22^ However, the functional role of FPR1 on RA pathogenesis and mode of action thereof remained to be resolved. Since we found that WKYMVm showed beneficial effects against CIA (Figure 1), and WKYMVm is a surrogate agonist for mouse FPR family members such as FPR1 and FPR2,^14^ we examined which FPR member is involved in WKYMVm‐induced anti‐CIA activity. We observed that the beneficial effects of WKYMVm against CIA and the suppressive activity of WKYMVm on T_H_1 and T_H_17 cell differentiation were selectively blocked by CsH but not by WRW4 (Figures 2, 3 and 4), suggesting a crucial role of FPR1 in the WKYMVm‐induced inhibition of CIA pathogenesis by regulating T_H_1 and T_H_17 cell differentiation, which is mediated by DCs, leading to beneficial effects against CIA. Our finding that therapeutic administration of WKYMVm effectively showed beneficial outcomes against CIA (Figure 6) strongly suggest the possibility of developing the peptide as a therapeutic agent to control RA in the future. One limitation of this study is that the functional role of FPR1 was only demonstrated by a pharmacological antagonist for the receptor. Further studies on the crucial roles of FPR1 and its human orthologue in the modulation of RA should be conducted in the near future.

In conclusion, we demonstrate that the activation of FPR1 on DC elicits beneficial effects against CIA. Mechanically, WKYMVm stimulates IL‐10 production from DCs via FPR1, and subsequently, IL‐10 contributes to the inhibitory effects on T_H_1 and T_H_17 cell generation, which are necessary for CIA progress. Our findings give rise to novel insight on the functional role of FPR1 on the inhibition of T_H_1 and T_H_17 under CIA pathogenesis and suggest that the immune regulating peptide WKYMVm is a useful material to control RA. We also suggest FPR1 as a major target to control DCs for the treatment of autoimmune diseases.

## CONFLICT OF INTEREST

Y.B and S.K. have a patent related to this study. The other authors confirm that there are no conflicts of interest.

## AUTHOR CONTRIBUTIONS

**Byunghyun Park:** Conceptualization (equal); Data curation (lead); Formal analysis (lead); Investigation (lead); Writing‐original draft (lead); Writing‐review & editing (equal). **Mingyu Lee:** Formal analysis (supporting); Investigation (supporting); Writing‐original draft (supporting). **Sang Doo Kim:** Conceptualization (supporting); Investigation (supporting). **Yu Sun Jeong:** Conceptualization (supporting); Investigation (supporting); Methodology (supporting). **Ji Cheol Kim:** Investigation (supporting); Methodology (supporting). **Siyoung Yang:** Conceptualization (supporting); Writing‐original draft (supporting); Writing‐review & editing (supporting). **Hye Young Kim:** Conceptualization (supporting); Writing‐original draft (supporting); Writing‐review & editing (supporting). **Yoe‐Sik Bae:** Conceptualization (lead); Funding acquisition (lead); Writing‐original draft (equal); Writing‐review & editing (equal).

## Data Availability

The data that support the findings of this study are available from the corresponding author upon reasonable request.
